# Crystal structure of (5*Z*)-5-(5-bromo-2-hy­droxy­benzyl­idene)-1,3-thia­zolidine-2,4-dione

**DOI:** 10.1107/S2056989015020654

**Published:** 2015-11-07

**Authors:** Joel T. Mague, Shaaban K. Mohamed, Mehmet Akkurt, Sabry H. H. Younes, Mustafa R. Albayati

**Affiliations:** a Department of Chemistry, Tulane University, New Orleans, LA 70118, USA; b Chemistry and Environmental Division, Manchester Metropolitan University, Manchester M1 5GD, England; c Chemistry Department, Faculty of Science, Minia University, 61519 El-Minia, Egypt; d Department of Physics, Faculty of Sciences, Erciyes University, 38039 Kayseri, Turkey; e Department of Chemistry, Faculty of Science, Sohag University, 82524 Sohag, Egypt; f Kirkuk University, College of Science, Department of Chemistry, Kirkuk, Iraq

**Keywords:** crystal structure, chalcones, thia­zolidinones, C—C bond formation, hydrogen bonding

## Abstract

In the title compound, C_10_H_6_BrNO_3_S, the dihedral angle between the thia­zolidine ring (r.m.s. deviation = 0.014 Å) and the benzene ring is 5.78 (14)°. The S atom of the heterocyclic ring is syn to the OH group attached to the benzene ring. In the crystal, inversion dimers linked by pairs of N—H⋯O hydrogen bonds generate *R*_2_^2^(8) loops. The dimers are linked into [001] ribbons by pairwise O—H⋯O hydrogen bonds with *R*_2_^2^(18) motifs. There are no short contacts involving the Br atom.

## Related literature   

For the biological activities of chalcones, see: Nowakowska (2007 ▸); Singh *et al.* (2011 ▸). For the various biological activities of thia­zolidinones, see: Cunico *et al.* (2008 ▸); Verma & Saraf, (2008 ▸); Hamama *et al.* (2008 ▸).
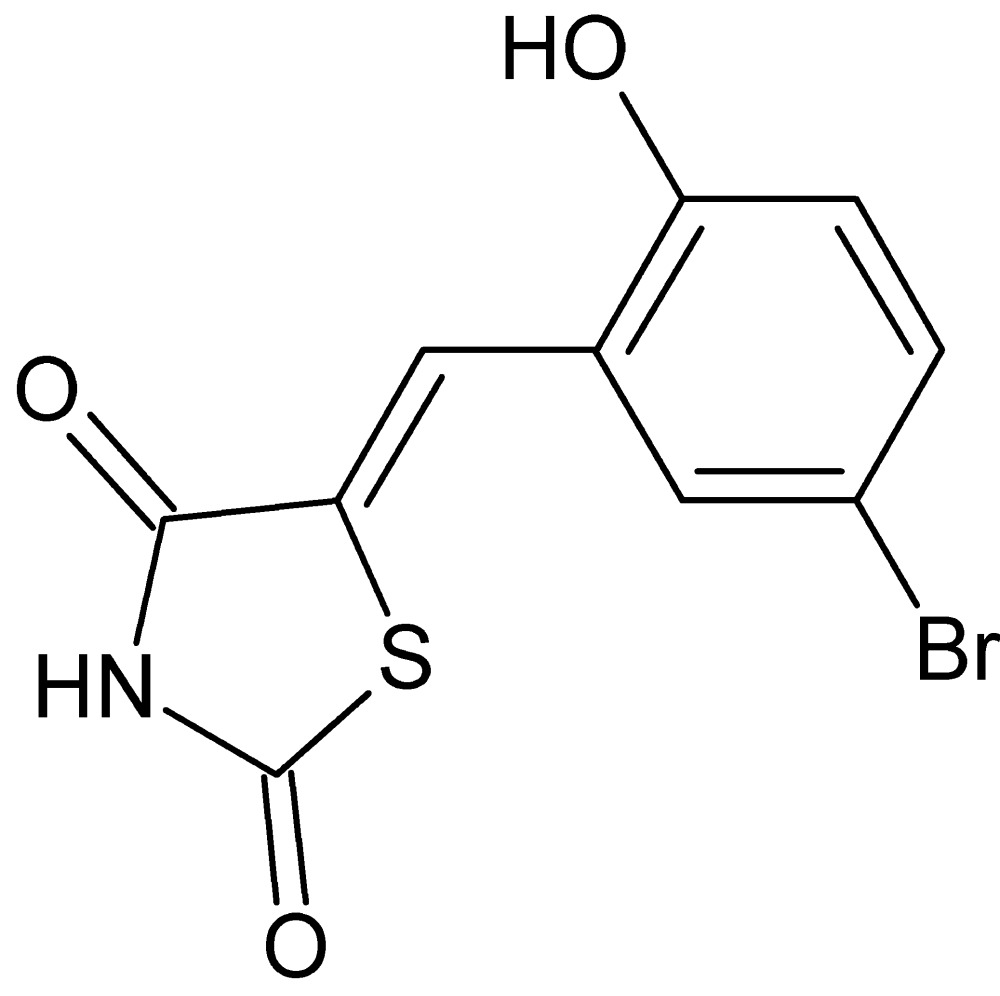


## Experimental   

### Crystal data  

C_10_H_6_BrNO_3_S
*M* *_r_* = 300.13
Triclinic, 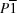

*a* = 7.0680 (7) Å
*b* = 7.6770 (8) Å
*c* = 9.9977 (10) Å
α = 68.119 (2)°
β = 86.049 (1)°
γ = 83.658 (1)°
*V* = 500.10 (9) Å^3^
*Z* = 2
Mo *K*α radiation
μ = 4.31 mm^−1^
*T* = 150 K
0.25 × 0.15 × 0.04 mm

### Data collection  

Bruker SMART APEX CCD diffractometer
Absorption correction: multi-scan (*TWINABS*; Sheldrick, 2015 ▸) *T*_min_ = 0.41, *T*_max_ = 0.85
25727 measured reflections
2629 independent reflections
2220 reflections with *I* > 2σ(*I*)
*R*_int_ = 0.060

### Refinement  

*R*[*F* ^2^ > 2σ(*F* ^2^)] = 0.033
*wR*(*F* ^2^) = 0.085
*S* = 1.01
2629 reflections
145 parameters
H-atom parameters constrained
Δρ_max_ = 0.82 e Å^−3^
Δρ_min_ = −0.77 e Å^−3^

### 

Data collection: *APEX2* (Bruker, 2015 ▸); cell refinement: *SAINT* (Bruker, 2015 ▸); data reduction: *SAINT* and *CELL_NOW* (Sheldrick, 2015 ▸); program(s) used to solve structure: *SHELXT* (Sheldrick, 2015 ▸); program(s) used to refine structure: *SHELXL2014* (Sheldrick, 2015 ▸); molecular graphics: *DIAMOND* (Brandenburg & Putz, 2012 ▸); software used to prepare material for publication: *SHELXTL* (Sheldrick, 2008 ▸).

## Supplementary Material

Crystal structure: contains datablock(s) global, I. DOI: 10.1107/S2056989015020654/hb7533sup1.cif

Structure factors: contains datablock(s) I. DOI: 10.1107/S2056989015020654/hb7533Isup2.hkl

Supporting information file. DOI: 10.1107/S2056989015020654/hb7533Isup3.cml

**Fig. 1.**
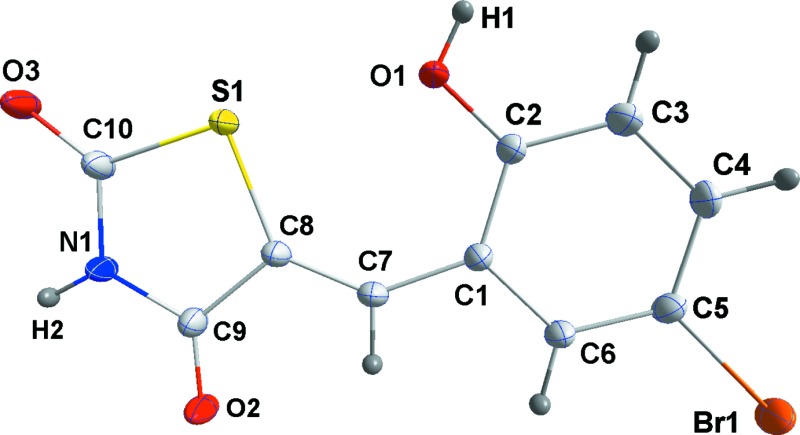
The title molecule with 50% displacement ellipsoids.

. DOI: 10.1107/S2056989015020654/hb7533fig1.tif
The title mol­ecule with 50% displacement ellipsoids.

**Fig. 2.**
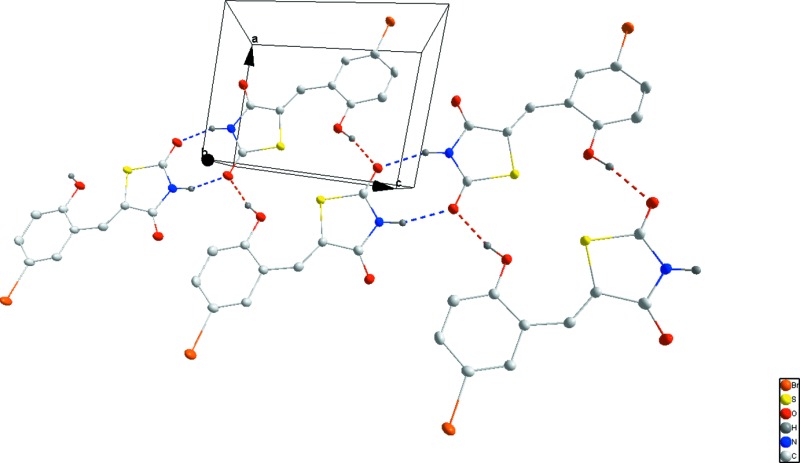
A portion of one layer generated by N—H···O and O—H···O hydrogen bonds (blue and red dotted lines respectively.

. DOI: 10.1107/S2056989015020654/hb7533fig2.tif
A portion of one layer generated by N—H⋯O and O—H⋯O hydrogen bonds (blue and red dotted lines respectively.

**Fig. 3.**
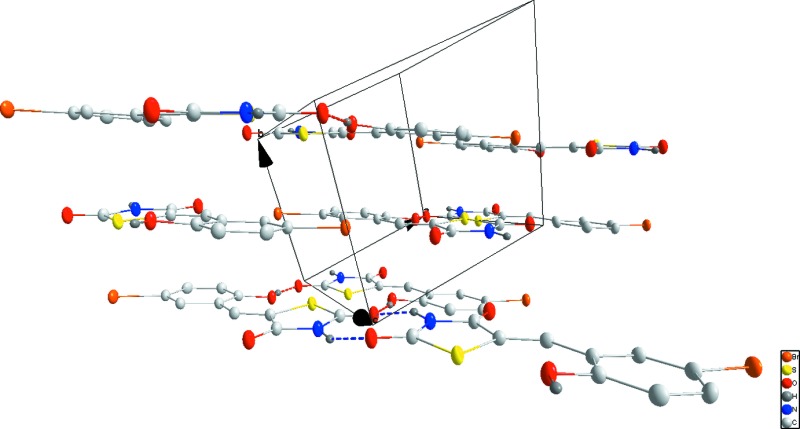
Elevation view of the layer structure with hydrogen bonds shown as in Fig. 2.

. DOI: 10.1107/S2056989015020654/hb7533fig3.tif
Elevation view of the layer structure with hydrogen bonds shown as in Fig. 2.

CCDC reference: 1434469

Additional supporting information:  crystallographic information; 3D view; checkCIF report

## Figures and Tables

**Table 1:** Hydrogen-bond geometry (Å, °)

*D*—H⋯*A*	*D*—H	H⋯*A*	*D*⋯*A*	*D*—H⋯*A*
O1—H1⋯O3^i^	0.84	1.91	2.740 (3)	168
N1—H2⋯O3^ii^	0.91	2.08	2.941 (3)	157

Symmetry codes: (i) 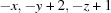; (ii) 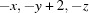.

## supplementary crystallographic information

### S1. Comment

Chalcones exhibit a wide spectrum of biological activities including antimicrobial, anticancer, anti-protozoal, antiulcer, and antiinflammatory ones (Nowakowska, 2007; Singh *et al.*, 2011). The tiazolidinone ring system has attracted the attention of many researchers to explore this skeleton to its multiple potential against several activities (Cunico *et al.*, 2008; Verma & Saraf, 2008; Hamama *et al.*, 2008). In this context we report here the synthesis and crystal structure of the title compound.

In the title molecule, the dihedral angle between the 6- and 5-membered rings is 5.8 (1)°. The molecules associate into dimers across centers of symmetry *via* pairwise N1—H2···O3 hydrogen bonds and these dimers associate with neighboring dimers through pairwise O1—H1···O3 hydrogen bonds across additional centers of symmetry to form ribbons (Fig. 2 and Table 1). Stacking of these ribbons generates the three-dimensional structure (Fig. 3).

### S2. Experimental

The title compound was obtained as a major product from a three component reaction of 5-bromo-2-hydroxy-benzaldehyde (1 mmol, 201 mg), thiazolidine-2,4-dione (1 mmol, 117 mg) and 1-aminopropan-2-ol (1 mmol, 75 mg) under reflux in 30 ml e thanol. The reaction was monitored by TLC till completion. On cooling the solid product was collected by filteration, dried under vacuum and recrystallized from ethanol to afford colourless plates. *M*.p. 503 K.

### S3. Refinement

Analysis of 1039 reflections having I/σ(I) > 13 and chosen from the full data set with *CELL_NOW* (Sheldrick, 2015) showed the crystal to belong to the triclinic system and to be twinned by a 180° rotation about c*. The raw data were processed using the multi-component version of *SAINT* under control of the two-component orientation file generated by *CELL_NOW*. H-atoms attached to carbon were placed in calculated positions (C—H = 0.95 - 0.99 Å) while those attached to nitrogen and to oxygen were placed in locations derived from a difference map and their coordinates adjusted to give N—H = 0.91 Å and O—H = 0.84 Å. All were included as riding contributions with isotropic displacement parameters 1.2 times those of the attached atoms. In the final stages of the refinement, runs using the full set of twinned data gave poorer results (in particular large residual peaks in the vicinity of Br1) than did the single-component data extracted with *TWINABS*. Consequently the refinement was completed with the single-component data.

### Crystal data

**Table:**

C_10_H_6_BrNO_3_S	*Z* = 2
*M**_r_* = 300.13	*F*(000) = 296
Triclinic, *P*1	*D*_x_ = 1.993 Mg m^−^^3^
*a* = 7.0680 (7) Å	Mo *K*α radiation, λ = 0.71073 Å
*b* = 7.6770 (8) Å	Cell parameters from 6176 reflections
*c* = 9.9977 (10) Å	θ = 2.9–29.0°
α = 68.119 (2)°	µ = 4.31 mm^−^^1^
β = 86.049 (1)°	*T* = 150 K
γ = 83.658 (1)°	Plate, colourless
*V* = 500.10 (9) Å^3^	0.25 × 0.15 × 0.04 mm

### Data collection

**Table:**

Bruker SMART APEX CCD diffractometer	2629 independent reflections
Radiation source: fine-focus sealed tube	2220 reflections with *I* > 2σ(*I*)
Graphite monochromator	*R*_int_ = 0.060
Detector resolution: 8.3333 pixels mm^-1^	θ_max_ = 29.1°, θ_min_ = 2.2°
φ and ω scans	*h* = −9→9
Absorption correction: multi-scan (*TWINABS*; Sheldrick, 2015)	*k* = −10→10
*T*_min_ = 0.41, *T*_max_ = 0.85	*l* = −12→12
25727 measured reflections	

### Refinement

**Table:**

Refinement on *F*^2^	Primary atom site location: structure-invariant direct methods
Least-squares matrix: full	Secondary atom site location: difference Fourier map
*R*[*F*^2^ > 2σ(*F*^2^)] = 0.033	Hydrogen site location: mixed
*wR*(*F*^2^) = 0.085	H-atom parameters constrained
*S* = 1.01	*w* = 1/[σ^2^(*F*_o_^2^) + (0.0497*P*)^2^] where *P* = (*F*_o_^2^ + 2*F*_c_^2^)/3
2629 reflections	(Δ/σ)_max_ = 0.001
145 parameters	Δρ_max_ = 0.82 e Å^−^^3^
0 restraints	Δρ_min_ = −0.77 e Å^−^^3^

### Special details

**Table:**

Experimental. The diffraction data were obtained from 3 sets of 400 frames, each of width 0.5° in ω, colllected at φ = 0.00, 90.00 and 180.00° and 2 sets of 800 frames, each of width 0.45° in φ, collected at ω = -30.00 and 210.00°. The scan time was 20 sec/frame.
Geometry. All e.s.d.'s (except the e.s.d. in the dihedral angle between two l.s. planes) are estimated using the full covariance matrix. The cell e.s.d.'s are taken into account individually in the estimation of e.s.d.'s in distances, angles and torsion angles; correlations between e.s.d.'s in cell parameters are only used when they are defined by crystal symmetry. An approximate (isotropic) treatment of cell e.s.d.'s is used for estimating e.s.d.'s involving l.s. planes.
Refinement. Refinement of *F*^2^ against ALL reflections. The weighted *R*-factor *wR* and goodness of fit *S* are based on *F*^2^, conventional *R*-factors *R* are based on *F*, with *F* set to zero for negative *F*^2^. The threshold expression of *F*^2^ > σ(*F*^2^) is used only for calculating *R*-factors(gt) *etc*. and is not relevant to the choice of reflections for refinement. *R*-factors based on *F*^2^ are statistically about twice as large as those based on *F*, and *R*- factors based on ALL data will be even larger. Analysis of 1039 reflections having *I*/σ(*I*) > 13 and chosen from the full data set with *CELL_NOW* (Sheldrick, 2008*a*) showed the crystal to belong to the triclinic system and to be twinned by a 180° rotation about c*. The raw data were processed using the multi-component version of *SAINT* under control of the two-component orientation file generated by *CELL_NOW*. H-atoms attached to carbon were placed in calculated positions (C—H = 0.95 - 0.99 Å) while those attached to nitrogen and to oxygen were placed in locations derived from a difference map and their coordinates adjusted to give N—H = 0.91%A and O—H = 0.84%A. All were included as riding contributions with isotropic displacement parameters 1.2 times those of the attached atoms. In the final stages of the refinement, runs using the full set of twinned data gave poorer results (in particular large residual peaks in the vicinity of Br1) than did the single-component data extracted with *TWINABS* (Sheldrick, 2015). Consequently the refinement was completed with the single-component data.

### Fractional atomic coordinates and isotropic or equivalent isotropic displacement parameters (Å^2^)

**Table:**

	*x*	*y*	*z*	*U*_iso_*/*U*_eq_	
Br1	1.07477 (3)	0.44030 (4)	0.79200 (3)	0.02259 (10)	
S1	0.14496 (8)	0.91252 (9)	0.38107 (7)	0.01849 (15)	
O1	0.2982 (2)	0.8558 (3)	0.6342 (2)	0.0209 (4)	
H1	0.2446	0.8745	0.7059	0.025*	
O2	0.5262 (2)	0.7995 (3)	0.1222 (2)	0.0241 (4)	
O3	−0.0849 (2)	1.0476 (3)	0.1603 (2)	0.0228 (4)	
N1	0.2180 (3)	0.9316 (3)	0.1180 (2)	0.0194 (5)	
H2	0.2025	0.9597	0.0224	0.023*	
C1	0.5808 (3)	0.7087 (4)	0.5672 (3)	0.0161 (5)	
C2	0.4680 (3)	0.7553 (4)	0.6732 (3)	0.0169 (5)	
C3	0.5335 (3)	0.7033 (4)	0.8128 (3)	0.0206 (5)	
H3	0.4545	0.7337	0.8833	0.025*	
C4	0.7120 (4)	0.6080 (4)	0.8501 (3)	0.0202 (5)	
H4	0.7557	0.5730	0.9452	0.024*	
C5	0.8263 (3)	0.5644 (4)	0.7449 (3)	0.0180 (5)	
C6	0.7631 (3)	0.6130 (3)	0.6081 (3)	0.0165 (5)	
H6	0.8437	0.5817	0.5387	0.020*	
C7	0.5313 (3)	0.7529 (4)	0.4200 (3)	0.0172 (5)	
H7	0.6347	0.7195	0.3656	0.021*	
C8	0.3763 (3)	0.8294 (3)	0.3389 (3)	0.0165 (5)	
C9	0.3887 (3)	0.8481 (4)	0.1850 (3)	0.0184 (5)	
C10	0.0738 (3)	0.9734 (4)	0.2026 (3)	0.0178 (5)	

### Atomic displacement parameters (Å^2^)

**Table:**

	*U*^11^	*U*^22^	*U*^33^	*U*^12^	*U*^13^	*U*^23^
Br1	0.01631 (13)	0.02877 (16)	0.02252 (17)	0.00492 (10)	−0.00595 (10)	−0.01027 (12)
S1	0.0135 (3)	0.0290 (3)	0.0140 (3)	0.0040 (2)	−0.0010 (2)	−0.0106 (3)
O1	0.0166 (8)	0.0310 (10)	0.0165 (10)	0.0072 (7)	−0.0018 (7)	−0.0126 (8)
O2	0.0184 (8)	0.0378 (11)	0.0164 (10)	0.0069 (8)	0.0002 (7)	−0.0132 (9)
O3	0.0162 (8)	0.0351 (11)	0.0194 (10)	0.0064 (8)	−0.0033 (7)	−0.0145 (9)
N1	0.0155 (9)	0.0303 (12)	0.0137 (11)	0.0044 (9)	−0.0017 (8)	−0.0113 (10)
C1	0.0134 (10)	0.0203 (12)	0.0152 (13)	0.0007 (9)	−0.0011 (9)	−0.0077 (10)
C2	0.0146 (10)	0.0215 (12)	0.0154 (13)	−0.0005 (9)	−0.0007 (9)	−0.0081 (10)
C3	0.0197 (12)	0.0248 (13)	0.0187 (14)	0.0003 (10)	0.0000 (10)	−0.0102 (11)
C4	0.0216 (11)	0.0240 (13)	0.0140 (13)	0.0009 (10)	−0.0043 (9)	−0.0059 (11)
C5	0.0144 (10)	0.0195 (12)	0.0203 (14)	0.0021 (9)	−0.0017 (9)	−0.0084 (11)
C6	0.0136 (10)	0.0201 (12)	0.0161 (13)	0.0013 (9)	−0.0003 (9)	−0.0080 (10)
C7	0.0160 (10)	0.0205 (12)	0.0164 (13)	0.0019 (9)	0.0010 (9)	−0.0094 (10)
C8	0.0150 (10)	0.0207 (12)	0.0142 (13)	0.0008 (9)	0.0009 (9)	−0.0076 (10)
C9	0.0159 (11)	0.0224 (13)	0.0169 (13)	0.0017 (9)	−0.0023 (9)	−0.0078 (11)
C10	0.0170 (11)	0.0231 (13)	0.0139 (13)	0.0018 (9)	−0.0012 (9)	−0.0084 (11)

### Geometric parameters (Å, º)

**Table:**

Br1—C5	1.903 (2)	C1—C7	1.441 (4)
S1—C10	1.762 (3)	C2—C3	1.398 (4)
S1—C8	1.770 (2)	C3—C4	1.387 (3)
O1—C2	1.350 (3)	C3—H3	0.9500
O1—H1	0.8399	C4—C5	1.399 (3)
O2—C9	1.220 (3)	C4—H4	0.9500
O3—C10	1.227 (3)	C5—C6	1.370 (4)
N1—C10	1.367 (3)	C6—H6	0.9500
N1—C9	1.391 (3)	C7—C8	1.352 (3)
N1—H2	0.9099	C7—H7	0.9500
C1—C2	1.411 (3)	C8—C9	1.489 (4)
C1—C6	1.416 (3)		
			
C10—S1—C8	91.56 (12)	C6—C5—Br1	119.73 (18)
C2—O1—H1	109.1	C4—C5—Br1	119.4 (2)
C10—N1—C9	116.6 (2)	C5—C6—C1	121.8 (2)
C10—N1—H2	121.1	C5—C6—H6	119.1
C9—N1—H2	122.3	C1—C6—H6	119.1
C2—C1—C6	117.0 (2)	C8—C7—C1	136.7 (2)
C2—C1—C7	126.5 (2)	C8—C7—H7	111.7
C6—C1—C7	116.5 (2)	C1—C7—H7	111.7
O1—C2—C3	121.5 (2)	C7—C8—C9	118.3 (2)
O1—C2—C1	117.9 (2)	C7—C8—S1	132.0 (2)
C3—C2—C1	120.6 (2)	C9—C8—S1	109.68 (17)
C4—C3—C2	121.1 (2)	O2—C9—N1	122.9 (2)
C4—C3—H3	119.5	O2—C9—C8	126.6 (2)
C2—C3—H3	119.5	N1—C9—C8	110.5 (2)
C3—C4—C5	118.6 (2)	O3—C10—N1	124.8 (2)
C3—C4—H4	120.7	O3—C10—S1	123.58 (19)
C5—C4—H4	120.7	N1—C10—S1	111.62 (18)
C6—C5—C4	120.9 (2)		
			
C6—C1—C2—O1	176.4 (2)	C1—C7—C8—C9	−179.6 (3)
C7—C1—C2—O1	−1.9 (4)	C1—C7—C8—S1	−1.4 (5)
C6—C1—C2—C3	−1.8 (4)	C10—S1—C8—C7	−179.7 (3)
C7—C1—C2—C3	179.9 (2)	C10—S1—C8—C9	−1.38 (19)
O1—C2—C3—C4	−176.9 (2)	C10—N1—C9—O2	178.0 (3)
C1—C2—C3—C4	1.3 (4)	C10—N1—C9—C8	−2.2 (3)
C2—C3—C4—C5	0.0 (4)	C7—C8—C9—O2	0.7 (4)
C3—C4—C5—C6	−0.6 (4)	S1—C8—C9—O2	−177.9 (2)
C3—C4—C5—Br1	178.16 (19)	C7—C8—C9—N1	−179.2 (2)
C4—C5—C6—C1	0.0 (4)	S1—C8—C9—N1	2.2 (3)
Br1—C5—C6—C1	−178.79 (18)	C9—N1—C10—O3	179.7 (2)
C2—C1—C6—C5	1.2 (4)	C9—N1—C10—S1	1.1 (3)
C7—C1—C6—C5	179.7 (2)	C8—S1—C10—O3	−178.4 (2)
C2—C1—C7—C8	−6.4 (5)	C8—S1—C10—N1	0.2 (2)
C6—C1—C7—C8	175.4 (3)		

### Hydrogen-bond geometry (Å, º)

**Table:**

*D*—H···*A*	*D*—H	H···*A*	*D*···*A*	*D*—H···*A*
O1—H1···O3^i^	0.84	1.91	2.740 (3)	168
N1—H2···O3^ii^	0.91	2.08	2.941 (3)	157

Symmetry codes: (i) −*x*, −*y*+2, −*z*+1; (ii) −*x*, −*y*+2, −*z*.
