# Supplementary figures and images for: Crystal structure of (5Z)-5-(5-bromo-2-hy­droxy­benzyl­idene)-1,3-thia­zolidine-2,4-dione
Source: Acta Crystallogr E Crystallogr Commun. 2015 Nov 7;71(Pt 12):o919–20. doi: 10.1107/S2056989015020654 (PMC4719880; doi:10.1107/S2056989015020654)

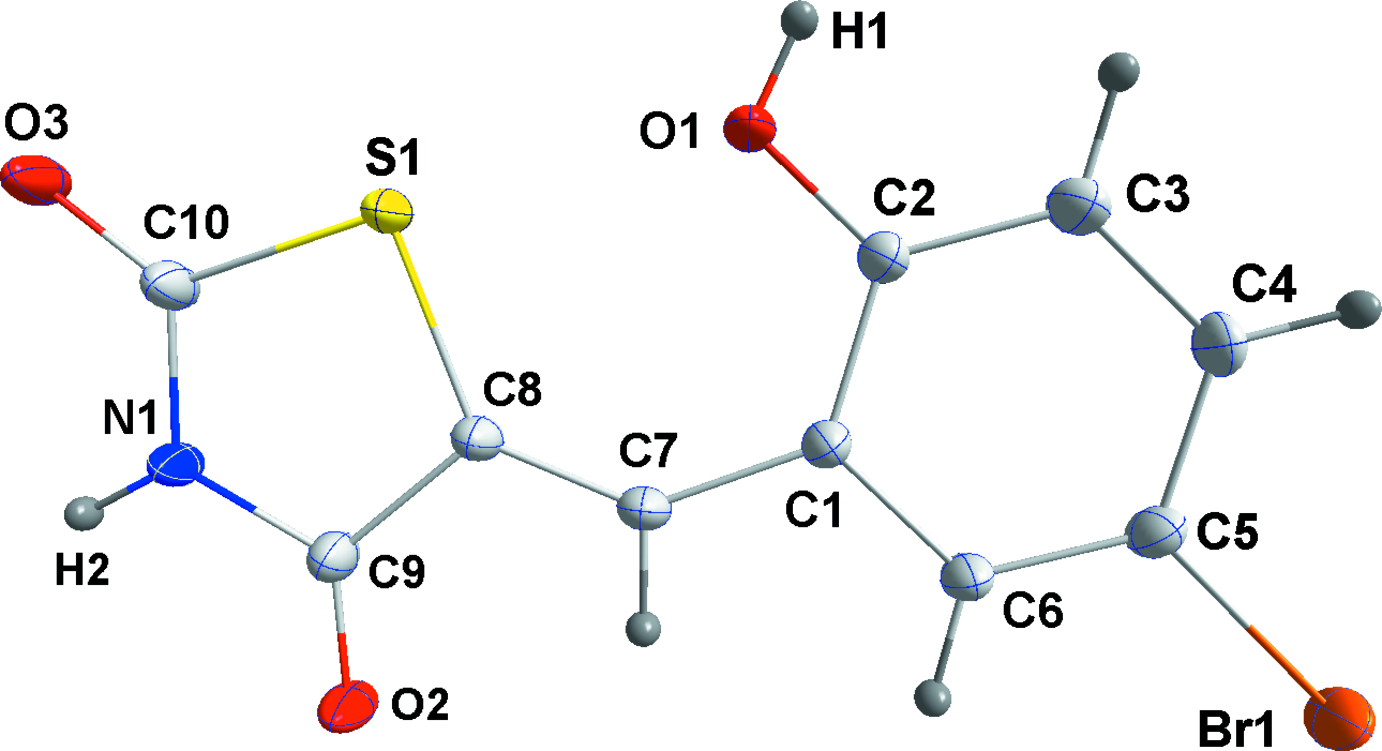

Supplement: Supplementary file 4 [file e-71-0o919-fig1.tif]

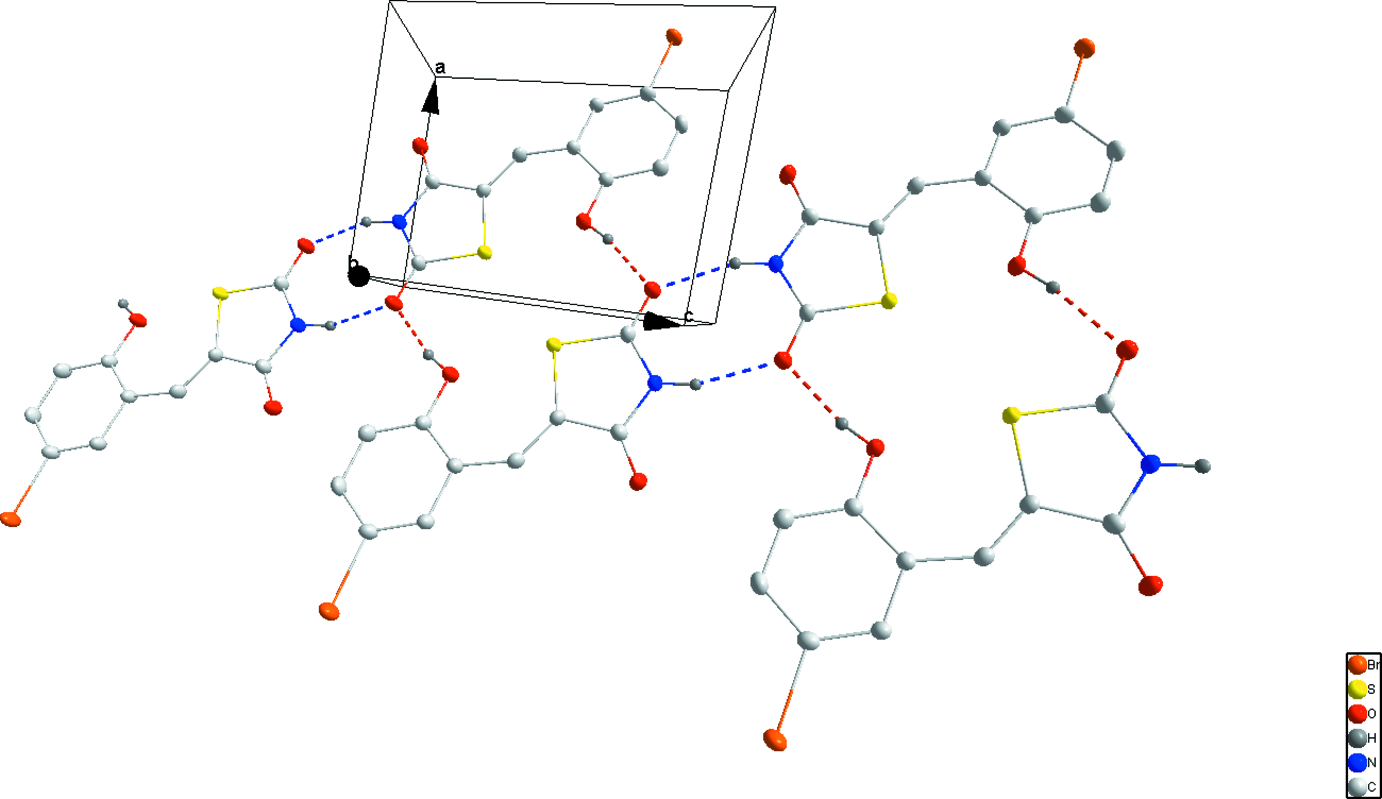

Supplement: Supplementary file 5 [file e-71-0o919-fig2.tif]

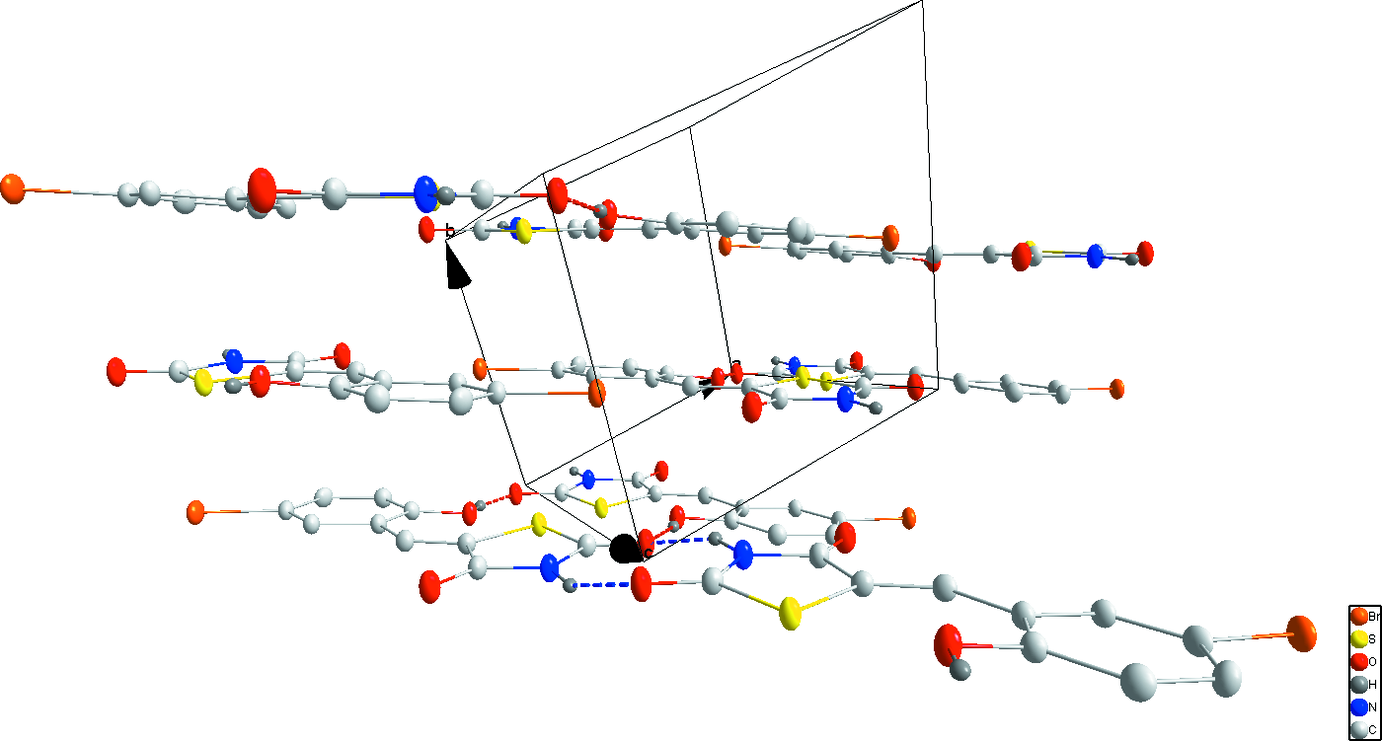

Supplement: Supplementary file 6 [file e-71-0o919-fig3.tif]
